# Estimating multiple morbidity disease burden among older persons: a convergent construct validity study to discriminate among six chronic illness measures, CCHS 2008/09

**DOI:** 10.1186/s12877-015-0001-8

**Published:** 2015-02-21

**Authors:** Andrew V Wister, Mélanie Levasseur, Lauren E Griffith, Ian Fyffe

**Affiliations:** Department of Gerontology, Simon Fraser University, 2800-515 Hastings Street, Vancouver, BC V6B 5K3 Canada; Research Centre on Aging, Health and Social Services Centre of the University Institute of Geriatrics of Sherbrooke, 1036 Belvédère sud, local 4427, Sherbrooke, QC J1H 4C4 Canada; School of Rehabilitation, Pavillon Gérald-Lasalle, local Z7-2524, Faculty of Medicine and Health Sciences Université de Sherbrooke, Sherbrooke, Canada; Department of Clinical Epidemiology & Biostatistics, Faculty of Health Sciences, McMaster University, Hamilton, Canada; Canadian Longitudinal Study on Aging (CLSA) Étude longitudinale canadienne sur le vieillissement (ÉLCV), Hamilton, Canada

**Keywords:** Multimorbidity indices, Surveys, Validation, Older adults

## Abstract

**Background:**

Since approximately two in three older adults (65+) report having two or more chronic diseases, causes and consequences of multimorbidity among older persons has important personal and societal issues. Indeed, having more than one chronic condition might involve synergetic effects, which can increase impact on disabilities and quality of life of older adults. Moreover, persons with multimorbidity require more health care treatments, implying burden for the person, her/his family and the health care system.

**Methods:**

Using the 2008/09 Canadian Community Health Survey (CCHS), this paper assesses the convergent construct validity of six measures of multimorbidity for persons aged 65 and over. These measures include: 1) *Multimorbidity Dichotomized (0, 1+ conditions)*; 2) *Multimorbidity Dichotomized (0/1, 2+);* 3) *Multimorbidity Additive Scale*; 4) *Multimorbidity Weighted by the Health Utility (HUI3) Scale*; 5) *Multimorbidity Weighted by the OARS Activity of Daily Living (ADL) Scale*; and 6) *Multimorbidity Weighted by HUI3 (using beta coefficients).* Convergent construct validity was assessed using correlations and OLS regression coefficients for each of the multimorbidity measures with the following social-psychological and health outcome variables: life satisfaction, perceived health, number of health professional visits, and medication use.

**Results:**

Overall, the two dichotomies (scales #1 & #2) showed the weakest construct validity with the health outcome variables. The additive chronic illness scale (#3) and the multimorbidity weighted by ADLs (#5), performed better than the other two weighted scales using (HUI #4 & #6). Measurement errors apparent in the dichotomous multimorbidity measures were amplified for older women, especially for life satisfaction and perceived health, but decreased when using the scales, suggesting stronger validity of scales #3 through #6.

**Conclusions:**

To properly represent multimorbidity, using dichotomous measures should be used with caution. When only prevalence data are available for chronic conditions, such as in the CCHSs or CLSA, an additive multimorbidity scale can better measure total illness burden than simple dichotomous or other discrete measures.

## Background

The study of the causes and consequences of multimorbidity has become highly important in recent years, especially among older persons given rapid population aging. Several interlocking trends explain such interest: approximately two in three seniors report having two or more chronic conditions, persons with multimorbidity require more health care treatments, and having more than one chronic illness may have synergetic detrimental (negative) effects [1-3]. Multimorbidity, the focus of the present study, is defined as conditions where an individual has been diagnosed with more than one chronic disease – a condition that is slow in progression, long in duration, and typically limits function, productivity and quality of life [4,5]. This can be distinguished from comorbidity, which also includes multiple chronic illnesses, but is defined in terms of an index disease, such as persons with cardiovascular disease who also have diabetes [6]. Although multimorbidity and comorbidity are overlapping terms, comorbidity tends to be used in research that focuses on one particular disease and a set of ‘secondary’ conditions, whereas multimorbidity simply includes all conditions that are present [4-6]. Although research within particular disease pillars (e.g., cardiovascular disease, cancer, arthritis, diabetes, etc.) has proliferated, a gap exists in the literature that addresses the simultaneous experience of living with multimorbidity.

Investigation into the etiology, trajectories, and outcomes of experiencing multimorbidity can help to understand total chronic illness burden and its risk factors [6,7]. Such understanding is important for several reasons, particularly, disentangling confounding (mediating or moderating) effects, and addressing the limitations of small numbers of cases for many illnesses found in secondary data sets, which reduces statistical efficiency [7,8]. In addition, and especially for older adults due to the trajectory of illness patterns across the age span, there may exist multiplicative or synergetic effects with the presence of multimorbidity that may be masked when grouping conditions together without taking into account specific numbers and differential impact of conditions. Whereas clinical studies of multimorbidity (or comorbidity) typically require detailed diagnostic data often from medical health records, such as type, onset and severity of illness, many population health surveys are restricted to self-reported prevalence data, thereby limiting public health research to simple measurement methods [9,10]. Given the availability of a growing number of national population health surveys internationally and in Canada, such as the Canadian Community Health Survey (CCHS) and the Canadian Longitudinal Study on Aging (CLSA), there is a need for better understanding of measurement approaches that tap into the complex public health issues of multimorbidity, including its causes and consequences. This paper seeks to fill this gap in knowledge by examining the convergent construct validity of six measures of multimorbidity for persons aged 65 and over using the 2008/09 CCHS - Healthy Aging survey.

While *comorbidity and multi-morbidity* are defined differently, there are common methodological approaches such that developments in one area can be applied to the other [7-9] In a systematic review, available methods to measure comorbidity published between 1966 and 2000 were compared and assessed with respect to their validity and reliability [7]. The majority used a list of defined diagnoses, disability level, and/or mortality risk scores in order to assign weights based on pathophysiologic severity ratings (i.e., symptoms, signs and laboratory tests) to individual illnesses. The authors found some degree of variability in the index scores, depending on the population and outcome used [7], suggesting the need to expand this research to examine other measures applied to different populations that can be used to help us understand aspects of public health.

Recently, a health-related quality of life (HRQL) comorbidity index (CI) was developed based on 44 clinical classification codes and weighted with beta coefficients derived from regressions on the Medical Outcome Study 12-Item Short-Form Health Survey (SF-12) physical health component and mental health components [1]. The HRQL-CI risk adjustment index performed better in terms of validity assessment than the Charlson-CI [7], a commonly used index that uses mortality risk weights in predicting general health status as well as for an asthma-specific (index disease) population. The development and validation of the HRQL-CI indicates that multi-faceted health function scales (such as those based on the SF-12 or SF-36) can be used to develop multimorbidity scales and for estimating total disease burden.

Another method of measuring the impact of multimorbidity was developed by calculating the effect of combinations of chronic diseases, typically those that are more common and severe, for key health outcomes such as functional status [2]. The disease combinations showed strong associations with the Older Americans Resources and Services (OARS) functional status measure. This approach proved to be useful in examining population attributable ‘functional status’ risk, a global disease burden measure developed using only prevalence data from the Canadian Study of Health and Aging first wave data. In addition, variations in the correlations with functional status occurred across age and gender groups, suggesting the need to incorporate these demographic variables in the construction of multimorbidity measures. A disease combination approach that does not weight the chronic illnesses has the advantage of a more direct interpretation, but the disadvantage of potentially omitting the impact of such conditions. Furthermore, prevalence estimates of multimorbidity have been shown to have considerable variability depending on the measure used in a systematic review of 21 articles [9].

The present study builds on previous work by examining the convergent construct validity of six measures of multimorbidity for persons aged 65 and over. Construct validity establishes the ability of an instrument to measure an abstract concept and the degree to which the instrument reflects the theoretical components of it. Convergent validity reflects the level in which two or more measures, that are believed to the same underlying phenomenon, will correlate highly [8]. Measures of multimorbidity included three commonly used non-weighted survey measures [10] (two dichotomies, and an additive scale using counts) and three constructed indexes where chronic illness categories are weighted based on HRQL and the OARS functional measure in order to reflect disease burden. These are subsequently validated comparatively using four key social-psychological and health outcomes. Older adults aged 65 and over were used in this study, since multimorbidity increases in prevalence in this age span and associations with health-related quality of life can differ across age groups [2].

## Methods

### The 2008/09 Canadian community health survey – healthy aging

The CCHS – Healthy Aging is a unique cross-sectional dataset commissioned by Statistics Canada as part of the CCHS program. From December 2008 to November 2009, 30,865 valid interviews were collected by way of a computer-assisted interviewing instrument. Approximately 94 per cent of interviews were conducted face-to-face by decentralized field interviewers who utilized the computer-assisted interviewing instrument. The remaining interviews were conducted over telephone due to extenuating circumstances. The target population were individuals aged 45 years or older in both rural and urban areas. Excluded from the sample frame were individuals: living within the three territories, living on First Nation reserves or Crown lands, living in institutions, living within some remote regions, or those who were employed full-time by the Canadian Forces. This study used the Public Use Sample File of the 2008/09 Canadian Community Health Survey available through the data liberation program of Canada. The original data was collected with full consent of all participants consistent with the Canada Statistics Act.

The sub-sample selected for this study includes 16,369 individuals aged 65 years or older. Weights generated by Statistics Canada were utilized in order to account for sampling error by age, gender and geographic region. The weighted sample was rescaled to the original sub-sample size so that the analyses were not overpowered statistically.

### Convergent construct validity with health outcomes: life satisfaction, perceived health, number of health professional visits and medication use

Construct validity refers to the degree to which a uniform test instrument or tool measures what it claims, or purports, to be measuring. Since there is no agreed upon gold standard criterion for examining the construct validity of multimorbidity, four separate social-psychological and health outcome variables being clinically important for measuring total disease burden were selected [11-26]. These include: ‘life satisfaction’, ‘perceived health status’, ‘number of health professional visits’, and ‘number of medications consumed on a daily basis’. Life satisfaction is a subjective cognitive appreciation of life that considers the expectations, reactions, and achievements of the individual, and has been found to be correlated with QOL and better health outcomes in old age [11-15]. Perceived health status has been found to be associated with QOL measures among community dwelling adults in the weak to moderate range [16-18]. An association between health care utilization, measures of multiple chronic illnesses and life satisfaction has also been reported in the literature [20]. Since health care utilization is a complex measure, we included a composite measure (total number of visits to health professionals), which was expected to be predicted by the presence of multimorbidity. Indeed, weighted multiple morbidity indexes have been shown to predict health care utilization [22]. Finally, medication use has also been used as a key outcome of illness presentation [23] and was therefore incorporated as the fourth validity criterion.

### Life satisfaction

This variable was measured using the Satisfaction with Life Scale (SWLS) [24]. The 5-item scale asked respondents to agree or disagree on a 7 seven-level Likert scale to the following questions: “in most ways my life is close to ideal”, “the conditions of my life are excellent”, “I am satisfied with my life”, “so far I have gotten the important things I want in life”, and “if I could live my life over, I would change almost nothing”. The SWLS mean score was 27.7 (s.d. = 5.2) with a range of 5–35, where higher scores indicate greater life satisfaction, and is comparable to other Canadian samples (see Table 1) [25]. A review and description of the psychometric properties of the SWLS including the convergent validity of the scale with a variety of health and social variables has been demonstrated in hundreds of studies [26].

**Table 1:** Chronic condition scales associations utilized for weighted scales creation (n = 16,369)*

**Chronic condition**	**Min/Max range HUI3 scale**	**ADL scale**	**HUI3 scale**
	**Pearson** ***r***	**Pearson** ***r***	**Standardized** β**
Arthritis	.27	.06	.16
Asthma	.07	.13	0
Back problems	.27	.11	.17
Blood pressure	.09	.06	0
Bowel disorder	.12	.10	.05
Bronchitis	.11	.06	.04
Cancer	.06	.02	.03
Cataracts	.11	.10	.04
COPD	.11	.07	.03
Diabetes	.12	.08	.07
Emphysema	.10	.06	.04
Glaucoma	.10	.10	.06
Heart disease	.16	.14	.08
Migraines	.11	.05	.05
Osteoporosis	.14	.13	.06
Stroke	.18	.20	.13
Thyroid condition	.03	.03	0
Ulcers	.10	.05	.03
Urinary incontinence	.22	.24	.14

*All associations are statistically significant at the p < .05 level at minimum, unless coded as 0.

**Controlled for age and gender using OLS regression modelling.

### Perceived health status

The self-perceived (self-reported) health measure employed in the CCHS Healthy Aging survey was based on a five point ordinal scale to the question: “In general, would you say your health is?” The response set was “poor”, “fair”, “good”, “very good” and “excellent”. This measure was highly positively skewed with a modal category of “good”. For the purpose of the correlation and multivariate analyses, we dichotomized this variable into fair/poor = 0, good/very good/excellent = 1.

### Number of health professional visits

This health care utilization measure was a derived variable created by the investigators of the CCHS. Prior to inquiring about the utilization of specific health care professionals the following question was asked “Now I’d like to ask about your contacts with various health professionals during the past 12 months, that is, from a year ago to yesterday”. This additive scale measured the number visits the respondent had during this time period for all health professionals including general practitioner, eye specialist, nurse, dentist, chiropractor, physiotherapist, occupational therapist, psychologist, social worker, audiologist, other specialist or other medical doctor. The mean response for this variable in this study was 2.85 (s.d. = 1.33) health professional visits within the past year.

### Medication use

This additive variable measured the number of medications consumed by a respondent on a daily basis based on self-reports. Individuals were asked whether or not they consume any of the following medication categories one at a time: pain relievers, tranquilizers, diet pills, anti-depressants, opioids, asthma medications, antibiotics, heart medicine, diuretics, steroids, sleeping pills, stomach remedies, laxatives, hormones, thyroid medicine or any other types of medicine. The mean number of daily medications reported in this study was 1.94, with a standard deviation of 1.52.

### Multiple morbidity measures and indexes

A total of 19 physical chronic conditions were available for inclusion in the CCHS: arthritis, asthma, back problems, blood pressure, bronchitis, cancer, cataracts, COPD, diabetes, emphysema, glaucoma, heart disease, migraine headaches, osteoporosis, stroke, thyroid condition, ulcers, and urinary incontinence. Due to their unique characteristics and similarly to other studies [27], mental health conditions were excluded.

Six multimorbidity measures were examined. Commonly used in health research, the first two measures utilized simple dichotomies as operationalizations of total chronic disease severity (considered to be crude multimorbidity count measures): 1) *Multimorbidity Dichotomized (0, 1+)* a dichotomized measure -- no chronic conditions versus one or more chronic conditions; and 2) *Multimorbidity Dichotomized (0/1, 2+)* a dichotomized measure -- 0 or 1 chronic conditions versus 2 or more. The next four measures produced unweighted and weighted scales: 3) *Multimorbidity Additive Scale* allowed the counts of chronic illnesses self-reported with each of the 19 illnesses was code 1 if present (range = 0 to 19); 4) *Multimorbidity Weighted by HUI3 Scale* weighted each chronic condition based on its correlation with the Health Utility Index (HUI3, see details below), then added these scores into a composite index; 5) *Multimorbidity Weighted by ADL Scale* used a similar procedure using the OARS functional status scale measuring instrumental activities of daily living; and finally, 6) *Multimorbidity Weighted by HUI3 (Betas) Scale* weighted each chronic condition using beta coefficients predicting HUI3 also adjusting for age and gender based on OLS regression modelling. Chronic illnesses with a non-statistically significant beta weight were scored zero in the scale. Table 1 presents the correlations and beta coefficients between the 19 chronic illnesses and the HUI3 and ADL scales that were used as weights to create measures 4 through 6. Table 2 presents descriptive statistics for all variables used in the subsequent analyses.

**Table 2:** **Descriptive statistics (n = 16, 369)**

**Interval/Dichotomous variables**	**Min/Max range**	**Mean**	**Standard deviation**
Multimorbidity (0, 1+)	0 to 1	.90	.30
Multimorbidity (0/1, 2+)	0 to 1	.71	.46
Multimorbidity Additive Scale	0 to 19	2.82	.43
Multimorbidity Weighted by HUI3	0 to 2.46	.43	.33
Multimorbidity Weighted by ADL	0 to 1.8	.28	.22
Multimorbidity Weighted by HUI3 betas	0 to 1.18	.21	.17
Number of health professional visits	0 to 10	2.85	1.33
Medication(s) used daily	0 to 11	1.94	1.52
Life satisfaction (5/35)	5 to 35	27.67	5.23
Perceived Health (0, 1)	0 to 1.00	.76	.42
HUI3 (−.32 /1)	-.32 to 1	.80	.24
OARS ADL Scale (0/8)	0 to 8	.53	1.27
**Ordinal and categorical variables**	**Categories**	**Frequency (%)**	
Age	65 to 69 years	4,949 (30.2)	
	70 to 74 years	4,074 (24.9)	
	75 to 79 years	3,254 (19.9)	
	80 to 84 years	2,249 (13.7)	
	85 and over	1,843 (11.3)	
Gender	Male	7,385 (45.1)	
	Female	8,984 (54.9)	

With respect to the two weighting variables used to create measures 4 through 6 above, the HUI3 is a broad-based multi-attribute measure of health-related quality of life and health status that has been utilized in hundreds of studies [27,28]. Eight dimensions of health status including vision, hearing, speech, ambulation, dexterity, emotion, cognition and pain are included in the scale. This sub-sample of older adults reported a mean HUI3 score of .80 and a standard deviation of .24, as well as a range of -.32 to 1.00. Scores below 0 on the HUI3 are indicative of a state worse than death. The HUI3 has been shown to have strong psychometric properties [28].

The performance in instrumental and basic activities of daily living scale (ADL scale) was estimated using a version of the OARS developed for use in the CCHS [29] which measures the ability to independently perform certain activities. These activities included: ‘use the phone’, ‘go places’, ‘go shopping’, ‘cook meals’, ‘do housework’, ‘take medicine’, ‘walk’, ‘bathe’ and ‘use the toilet’. The minimum score of this scale was 0 and the maximum was 8, while the mean score and standard deviation was .53 and 1.27, respectively. The OARS has been proven to have strong test-retest reliability and criterion validity [29]. Both the HUI3 and the ADL scales are useful as weighting variables, since HUI3 captures multiple dimensions of health-related quality of life in health, and the ADL scale measures one’s ability to perform specific tasks.

### Statistical analysis

Individually, correlations of each measure was examined with the four health outcome variables used as criteria. While there are a number of ad hoc cut-offs for assessing strength of correlation statistics, we employed the correspondence rule whereby associations under .29 indicated poor convergent validity; those between of .30 to .49 provided evidence of moderate levels; and .50 and over indicted strong levels [30]. In addition, comparatively, the correlations for each of the scales were compared in relation to the *Multimorbidity Dichotomized 0/1+* (base reference measure), which compared individuals without chronic condition with those who had one or more chronic illnesses. We selected this measure as the base comparison measure because it has been widely used as a measure of chronic illness and multimorbidity, it was the most simplistic, and it produced the lowest correlations with the health outcome variables. To assess each multimorbidity measure, the percentage increase in correlational strength with the outcome variables was calculated for the five other multimorbidity measures compared to the variable *Multimorbidity Dichotomized*. It should be noted that the percentage increases in the correlations for the five multimorbidity variables and the outcome variables compared to the reference dichotomous measure vary according to the correlations for the latter. For example, when the correlation for the reference measure and an outcome variable is low, percentage increases for the comparison measures tend to be higher. It is therefore important to examine the absolute size of the correlations in conjunction with the percentage increase compared to the base or reference measure. Moreover, to objectively verify equality of correlation coefficients among the six multimorbidity measures, we used Olkin’s test for single samples [31].

where,

$$ Z{=}_{\sqrt{{\left(1 - {r}_{12}^2\right)}^2 + {\left(1 - {r}_{12}^2\right)}^2 - 2{r}_{23}^3 - \left(2{r}_{23} - {r}_{12\ }\ {r}_{13}\right)\left(1 - {r}_{12\kern0.75em }^2-\kern0.5em {r}_{13\kern0.75em }^2-\kern0.5em {r}_{23}^2\right)}}^{\kern13em \left({r}_{12 - }{r}_{13}\right)\ \sqrt{n}} $$

Given that prior research has demonstrated that some multimorbidity measures used to estimate population attributable risk vary according to age and gender [2], we also examine age and gender difference in these associations.

## Results

The 65 and over sub-sample (n = 16,369) used for this analysis included slightly more females (54.9%) than males (45.1%); and more participants aged 75 and over (57.4%) than 65 to 74 (42.6%). The sample was highly educated, with almost half (42.8%) who reported an education level greater than secondary school graduation. In addition, most of the sample reported being married or in a common-law relationship (63.4%), followed by widowed (25.4%), divorced or separated (7.4%), and single (3.8%) categories. Generally, a majority self-reported ‘good’, ‘very good’ or ‘excellent’ health (76.5%). Also, more than three quarters (77.4%) reported no functional impairments in performing instrumental activities of daily living including meal preparation, bathing, and being able to walk without help.

### Bivariate analysis

Table 3 presents bivariate correlations between each of the six multimorbidity measures and the four outcome variables; percentage change increase in correlations (across rows) using the *Multimorbidity Dichotomized 0/1+* measure as the reference category (shown in parentheses); and a matrix of statistical tests among all of the correlations using Olkin’s test (shown with letter subscripts and footnotes). Comparisons among the six scales and the health outcome variables were all statistically significant at the p < .001 level (Table 3), but differences in strength of associations with the health outcome variables were observed. Overall, all of the scales that either added all chronic illnesses, or added them using weights, presented stronger associations than either the simple *Multimorbidity Dichotomized 0/1+* or the *Multimorbidity Dichotomized 0,1/2+* measure (Table 3. For instance, using either dichotomous measure resulted in poor convergent validity with life satisfaction (*r =* .10 and .14, respectively), number of health professional visits (*r =* .14 and .19, respectively) and for perceived health (*r =* .15 and .25, respectively); and only moderate levels for medication use (*r =* .30 and .42, respectively). The dichotomous measure using 0/1 and 2+ categories of chronic illnesses resulted in modestly stronger correlations with the outcome variables than the basic 0 vs. 1+ dichotomous measure (Table 3). The four additive scales (with or without being weighted to HUI3 or ADLs) exhibited consistently moderate to strong construct validity (ranging between *r =* .21 and .55). Interestingly, the *Multimorbidity Weighted by HUI3 (Beta) Scale* that controlled for age and gender, did not perform better than the other weighted scales (Table 3). The percentage increase in the correlations between the four additive chronic illness scales (weighted or unweighted) and the dichotomous measures ranged between 130% and 160% for life satisfaction and perceived health outcomes, and between 35.7% and 83.3% for number of health professional visits and daily medication use. We also used the full five-point ordinal measure of perceived health (calculating gamma scores) with no differences in results in the strength and significance of the associations reported (not reported in tables). In addition, analyses were repeated using transformations (square root and log 10, adjusting for zero scores) of the skewed outcomes (medication use, healthcare utilization, and life satisfaction) to normalize their distributions. These supplementary analyses resulted in negligible differences between .01 - .03 (not reported in tables).

**Table 3:** Bivariate correlation coefficients between multimorbidity measures and health outcomes,* relative percentage change in coefficients,** and matrix of intercorrelation statistical tests of associations*** (n = 16,369)

	**Multi-morbidity dichotomized (0, 1+) (Reference)**	**Multi-morbidity dichotomized (0/1, 2+)**	**Multi-morbidity additive scale**	**Multi-morbidity weighted by HUI3**	**Multi-morbidity weighted by ADL**	**Multi-morbidity weighted by HUI3 (Betas)**
Life Satisfaction	-.10^a^	-.14^a^ (40.0)*	-.23^b^ (130.0)	-.24^b^ (140.0)	-.24^b^ (140.0)	-.24^b^ (140.0)
Perceived Health	-.15^a^	-.25^a^ (66.7)	-.39^b^ (160.0)	-.39^b^ (160.0)	-.39^b^ (160.0)	-.39^b^ (153.3)
Health Prof. Visits	.14^a^	.19^a^ (35.7)	.22^b^ (57.1)	.22^b^ (57.1)	.22^b^ (57.1)	.21^b^ (50.0)
Medication(s) Used Daily	.30^a^	.42^a^ (40.0)	.55^a^ (83.3)	.48^c^ (60.0)	.50^d^ (66.7)	.43^e^ (43.3)

*All correlations are statistically significant at the p < .001 level.

**Values in parentheses represent percentage increase in strength of association between multimorbidity measures and health outcome (rows) using the Multimorbidity Dichotomized (0, 1+) measure as the reference.

***Intercorrelation matrix notes:

^a^Correlation differs significantly from all other multimorbidity measures.

^b^Correlation only differs significantly from the two dichotomous multimorbidity measures.

^c^Correlation differs significantly from all other multimorbidity measures, except Multimorbidity Weighted by ADL.

^d^Correlation differs significantly from all other chronic illness measures, except Multimorbidity Weighted by HUI.

^e^Correlation differs significantly from all other chronic illness measures, except Multimorbidity Dichotomized (0/1,2+).

As shown in Table 3 the strongest absolute correlations with the four outcome variables were found for the additive and weighted scales and medication use (ranged between *r =* .42 and .55), as well as for perceived health (*r =* .39 for all scales). The similarity in the correlations for perceived health may be due to using a dichotomy for this outcome variable. In addition, the *Multimorbidity Additive Scale* produced the highest percentage increases on the four health outcome variables in comparison to the dichotomized (0/1+ illnesses) reference scale using bivariate correlational analysis techniques. However, it should be noted that this scale only performed marginally better than the *Multimorbidity Weighted by ADL Scale*; the *Multimorbidity Weighted by HUI3* correlational scale, and the *Multimorbidity Weighted by HUI3 (Betas) Scale* (Table 3. In addition, the chronic conditions dichotomized scale (0/1, 2+) performed marginally better than the simple dichotomy using 0 vs. 1+ base measure.

Table 3 also presents a matrix of statistical tests (p < .001) of comparisons among all correlation coefficients of the chronic illness measures and the separate outcome variables (see alphabetical subscripts). The correlations between each of the dichotomous illness variables and all other chronic illness measures (including the unweighted and weighted scales) were statistically different, given the large differences in the size of the correlations. However, comparisons among only the four additive and weighted multimorbidity scales were *not* statistically significant, except in the instance of medication use. Statistical tests comparing correlations between the multimorbidity measures and the outcomes was not reported for the subsequent analyses due to replication of results.

### Linear regression analysis

Controlling for age and gender, comparison of the six scales in association with the health outcome variables (Table 4 replicated the bivariate correlation findings (Table 3). However, exceptions were found for some small differences in the ranking of the four additive and weighted scales in terms of strength of convergent validity. In addition, these four scales exhibited larger percentage increases in associations with the health outcome variables compared to the simple dichotomy using 0/1+ illness (reference group). Overall, using linear regression modelling techniques, the *Multimorbidity Weighted by ADL Scale* and the *Multimorbidity Additive Scale* produced the highest increases on the four health outcome variables in comparison to the *Multimorbidity dichotomized* (0/1+) base reference scale. With respect to individual health outcome variables, the strongest correlations were again found for medication use and perceived health (Table 4). A matrix of statistical tests comparing intercorrelations among the multimorbidity measures and the outcomes replicated the findings in Table 3 and were therefore omitted from Tables 4, 5, and 6.

**Table 4:** Standardized beta coefficients of associations between multimorbidity measures and health outcome,* controlling for age and gender, and relative percentage change in coefficients** (n = 16,369)

	**Multi-morbidity dichotomized (0, 1+) (Reference)**	**Multi-morbidity dichotomized ( 0/1, 2+)**	**Multi-morbidity additive scale**	**Multi-morbidity weighted by HUI3**	**Multi-morbidity weighted by ADL**	**Multi-morbidity weighted by HUI3 (Betas)**
Life Satisfaction	-.09	-.14 (55.6)	-.24 (166.7)	-.25 (177.8)	-.25 (177.8)	-.25 (177.8)
Perceived Health	-.15	-.24 (60.0)	-.40 (166.7)	-.39 (160.0)	-.40 (166.7)	-.37 (146.7)
Health Prof. Visits	.14	.20 (42.9)	.23 (64.3)	.23 (64.3)	.24 (71.4)	.23 (64.2)
Medication(s) Used Daily	.28	.40 (43.0)	.55 (96.4)	.48 (20.0)	.50 (78.6)	.42 (50.0)

*All values are significant at the p < .001 level.

**Values in parentheses represent percentage increase in strength of association between multimorbidity measures and health outcome (rows) using the Multimorbidity Dichotomized (0, 1+) measure as the reference.

**Table 5:** Bivariate correlation coefficients of associations between multimorbidity measures and health outcome variables,* 65–74 by gender, and relative percentage change in coefficients** (n = 6,975)

	**Multimorbidity dichotomized (0, 1+) (Reference)**		**Multimorbidity dichotomized (0/1, 2+)**		**Multimorbidity additive scale**		**Multimorbidity weighted by HUI3 scale**		**Multimorbidity weighted by ADL scale**		**Multimorbidity weighted by HUI3 (Betas) scale**	
**Gender**	**Men**	**Women**	**Men**	**Women**	**Men**	**Women**	**Men**	**Women**	**Men**	**Women**	**Men**	**Women**
Life Satisfaction	-.13	-.07	-.18 (38.5)	-.13 (85.7)	-.27 (107.7)	-.24 (242.9)	-.28 (115.4)	-.25 (257.1)	-.27 (107.7)	-.25 (257.1)	-.27 (107.7)	-.24 (242.9)
Perceived Health	-.19	-.14	-.30 (57.9)	-.26 (85.7)	-.42 (121.1)	-.42 (200.0)	-.39 (105.3)	-.41 (192.9)	-.40 (110.5)	-.42 (200.0)	-.37 (94.7)	-.39 (178.6)
Health Prof. Visits	.17	.11	.20 (17.6)	.19 (72.7)	.22 (29.4)	.20 (81.8)	.21 (23.5)	.20 (81.8)	.22 (29.4)	.21 (90.9)	.19 (11.8)	.19 (72.7)
Medication Used Daily	.33	.26	.46 (39.4)	.39 (50.0)	.54 (63.6)	.55 (111.5)	.46 (39.4)	.47 (80.8)	.51 (54.5)	.49 (88.5)	.38 (15.2)	.42 (61.5)

*All values are significant at the p < .001 level.

**Values in parentheses represent percentage increase in strength of association between multimorbidity measures and health outcome (rows) using the Multimorbidity Dichotomized (0, 1+) measure as the reference.

**Table 6:** Bivariate correlation coefficients of associations between multimorbidity measures and outcome variables,* 75+ by gender, and relative percentage change in coefficients** (n = 9,394)

	**Multimorbidity dichotomized (0, 1+) (Reference)**		**Multimorbidity dichotomized (0/1, 2+)**		**Multimorbidity additive scale**		**Multimorbidity weighted by HUI3 scale**		**Multimorbidity weighted by ADL scale**		**Multimorbidity weighted by HUI3 (Betas) scale**	
**Gender**	**Men**	**Women**	**Men**	**Women**	**Men**	**Women**	**Men**	**Women**	**Men**	**Women**	**Men**	**Women**
Life Satisfaction	-.10	-.07	-.13 (30.0)	-.10 (42.9)	-.19 (90.0)	-.21 (200.0)	-.21 (110.0)	-.23 (185.7)	-.21 (110.0)	-.23 (185.7)	-.20 (100.0)	-.23 (185.7)
Perceived Health	-.16	-.09	-.23 (43.8)	-.18 (100.0)	-.36 (125.0)	-.36 (300.0)	-.35 (118.8)	-.38 (322.2)	-.36 (125.0)	-.37 (311.1)	-.33 (106.3)	-.37 (311.1)
Health Prof. Visits	.17	.09	.21 (23.5)	.16 (77.8)	.28 (64.7)	.23 (155.6)	.28 (64.7)	.22 (144.4)	.27 (58.8)	.23 (155.6)	.26 (52.9)	.21 (133.3)
Medication Used Daily	.32	.26	.42 (31.3)	.35 (34.6)	.51 (59.4)	.55 (111.5)	.46 (43.8)	.48 (84.6)	.47 (46.9)	.49 (88.5)	.40 (25.5)	.43 (65.4)

*All values are significant at the p < .001 level.

**Values in parentheses represent percentage increase in strength of association between multimorbidity measures and health outcome (rows) using the Multimorbidity Dichotomized (0, 1+) measure as the reference.

### Bivariate analysis by age and gender

Age groups: 65 to 74 and 75+, and gender analyses (Tables 5 and 6), largely replicate the above results. The size of the correlations for the additive and weighted multimorbidity scales and the outcome variables are considerably higher (approximately twice as large) than for the two dichotomous measures. The only exceptions are for number of health professional visits among both age groups, and daily medication use for the 75+ age group, where the *Multimorbidity Dichotomized (0/1, 2+)* measure approaches the strength of the correlations for the additive and weighted multimorbidity scales. Overall, the *Multimorbidity Additive Scale* and the *Multimorbidity Weighted by ADL Scale* result in the largest correlations with the four health outcome variables, and the largest increases in correlational strength relative to the simple 0,1+ dichotomous chronic illness measure. This occurred for both genders and both age groups (Tables 5 and 6), but with some degree of variability depending on the outcome measure, age group and gender. For instance, among women aged 65–74, the percentage increase in correlational strength for these two scales compared to the reference scale ranged between 81.8% for the additive scale (number of health professional visits) and 257.1% for the weighted ADL scale (life satisfaction). In addition, among all age-gender groups, the absolute correlations with the outcome variables were largest for perceived health and daily medication use.

One statistical artifact is important to note. Since the correlations between the reference scale (dichotomized 0/1+ illnesses) and the health outcome variables was lower for the women than men (Tables 5 and 6), the percentage increases in the correlations for the multimorbidity scales were two to three times larger for the women than for the men, even though the absolute strength of the correlations did not vary across gender. This pattern was observed for both the 65–74 and the 75+ age categories (Tables 5 and 6). Yet, when the more robust additive scales are used, whether unweighted, or weighted to HUI (a measure of HRQL) or ADLs (a measure of functional status), the differences between correlations of men and women were attenuated. With respect to individual health outcome variables, the strongest correlations appeared for medication use and perceived for older women than for older men.

## Discussion

In an effort to better estimate multimorbidity and its public health burden, this paper examined the convergent construct validity of six measures of multimorbidity with selected health outcomes, i.e., a life satisfaction scale, perceived health, number of health professional visits, and daily medication use. Two of these were simple dichotomous variables using 0/1+ and 0,1/2+ coding of the 19 chronic illnesses available in the CCHS, with the first used as a base reference measure to assess the others. The other four were additive scales and included: 1) a scale in which the 19 chronic illnesses were counted (each receiving a 1 when reported by a respondent); 2) a scale weighted according to individual correlations with the Health Utility Index (HUI3), a global health quality of life measure (HRQL); 3) a scale weighted using the OARS ADLs, a measure of functional status or disability; and 4) a scale weighted using betas generated from an OLS regression using HUI3 as the health outcome variable, and all 19 chronic illnesses entered as predictors, after age and gender were included.

The simple additive scale using counts of illnesses reported (prevalence) and the weighted scale using correlations between each of the chronic illnesses and the OARS ADLs scale produced the strongest and most consistent construct validity with the four health outcome variables. Yet, the absolute and relative increases in the associations between the multimorbidity measures and the health outcomes showed a clear and striking pattern of stronger convergent validity for *all* four of the additive scales whether weighted or unweighted, compared to the dichotomous multimorbidity measures. These findings remained robust when statistically controlling for age and gender using OLS regression, and when analysis was conducted for two age groups (65–74 and 75+) and for women and men separately. However, in part, since the correlations for the base reference scale (dichotomized 0/1+) were smaller for women, the percentage increase in correlations were twice as large for older women than for older men. Such gender difference in the correlations disappears when using the four additive scales, whether unweighted or weighted using HUI or ADLs. Indeed, when using simple dichotomous multimorbidity measures, errors in estimating the effect of multimorbidity on health outcomes are magnified for older female populations, whereas more sophisticated scales eliminates these gender differences. This further suggests that the four additive scales have stronger convergent construct validity than the simple dichotomies.

Research into the health of an aging population needs to reflect on the best ways to consider multimorbidity measures. Although there are many secondary datasets sets (e.g., CCHSs, CSHA, CLSA, etc.) containing health data with illness prevalence, there is often no detail on other dimensions found in medical records (i.e., onset, severity, prognosis, etc.). Researchers interested in a global measure of public health burden based on multimorbidity, or utilizing a multimorbidity measure as predictor, mediator or moderating variable, should be careful of using dichotomous measures that split chronic illnesses into 0 and 1 or more, or 0/1 and 2 or more. Indeed, like most continuous variables, a simple additive scale or count of illnesses performs considerably better than either of the former. Among weighted measures, the one that uses the OARS ADLs scale is preferred over HUI3. However, the weight functions did not add significantly to the scales generated using these techniques. This finding is important because researchers can employ the easily calculated additive multimorbidity scale rather than more complex weighted scales without compromising rigor. Such finding also suggests that weighting chronic illnesses to functional status scales (based on HUI3 & ADL dimensions) does not significantly improve prediction of subjective health (life satisfaction & perceived health), health professional visits, and medication use, over and above counting the number of chronic illnesses and assigning them equal weight. Research that examines specific combinations of chronic illnesses may also shed light on the health impact of multimorbidity using different measures. Furthermore, we discovered that using simple dichotomies to estimate the effects of multimorbidity will significantly underestimate its association with specific health outcomes, as well as underestimate the disease burden connected to presenting with multimorbidity.

This study is the first of its kind to use a large representative sample of older community-living Canadians to compare measures of multimorbidity. Given the proliferation of studies examining the effects of multimorbidity on disease burden, heath care utilization, and HRQL, increased understanding of the implications of selecting different measures of multimorbidity from survey instruments will help to guide research. Indeed, the results of this study provide definitive conclusions pertaining to the choice of measure used to estimate multimorbidity. It is not uncommon for many published studies to use inferior measures of multimorbidity, a decision that may be seriously biasing results.

This research is limited by the available measures in the CCHS. For instance, the Health Utility Index measure was used in lieu of others (i.e. SF36/12). In addition, the four health outcome variables selected for the construct validity analysis are not exhaustive of all relevant measures. For example, measures of psychological well-being, social isolation, and medical diagnostic information may prove to be useful for further study. Future research needs to examine whether a subset of chronic illnesses (such as heart disease, cancer, arthritis, diabetes, and COPD) would adequately estimate multimorbidity as a measure of global disease burden or population attributable risk [2]. Nevertheless, the four multimorbidity scales demonstrated moderate to strong correlations with the health outcomes in this study. Furthermore, research needs to investigate other algorithms to differentially weight multimorbidity measures. While the authors also used a log + 1 formulae to convert beta scores, and applied beta weights using the ADL measure without improving the performance of the measure (not shown in this paper), other techniques need to be examined. Application to other age or cultural groups may also be informative. More sophisticated weighting techniques based on diagnostic criteria (e.g., illness onset, severity, etc.) or mortality data, may further add to this body of research. Finally, the statistical differences using Olkin’s (1964) formula should be interpreted with care, since the sample size was large, resulting in small correlational differences in the order of approximately .04 being statistically significant at the p < .001 level.

## Conclusion

The increasing numbers of older persons and the presentation of more complex constellations of chronic diseases has contributed to a growth in research examining burden of multimorbidity. This paper recommends that health researchers interested in a multimorbidity measure using survey data, such as those available in the CCHSs or the CLSA for older populations should utilize either an additive scale or one weighted using a disability measure such as the OARS ADLs scale. More research is warranted to improve knowledge in the development and comparative analysis of multimorbidity measures and outcomes.
